# Catalytic Metal Nanoparticles Embedded in Conductive Metal–Organic Frameworks for Chemiresistors: Highly Active and Conductive Porous Materials

**DOI:** 10.1002/advs.201900250

**Published:** 2019-09-12

**Authors:** Won‐Tae Koo, Sang‐Joon Kim, Ji‐Soo Jang, Dong‐Ha Kim, Il‐Doo Kim

**Affiliations:** ^1^ Department of Materials Science and Engineering Korea Advanced Institute of Science and Technology (KAIST) 291 Daehak‐ro Yuseong‐gu Daejeon 34141 Republic of Korea; ^2^ Advanced Nanosensor Research Center KI Nanocentury Korea Advanced Institute of Science and Technology (KAIST) 291 Daehak‐ro Yuseong‐gu Daejeon 34141 Republic of Korea; ^3^ Present address: Department of Materials Science and Engineering Massachusetts Institute of Technology Cambridge MA 02139 USA

**Keywords:** catalysts, conductive, metal nanoparticles, metal–organic frameworks, sensors

## Abstract

Conductive porous materials having a high surface reactivity offer great promise for a broad range of applications. However, a general and scalable synthesis of such materials remains challenging. In this work, the facile synthesis of catalytic metal nanoparticles (NPs) embedded in 2D metal–organic frameworks (MOFs) is reported as highly active and conductive porous materials. After the assembly of 2D conductive MOFs (C‐MOFs), i.e., Cu_3_(hexahydroxytriphenylene)_2_ [Cu_3_(HHTP)_2_], Pd or Pt NPs are functionalized within the cavities of C‐MOFs by infiltration of metal ions and subsequent reduction. The unique structure of Cu_3_(HHTP)_2_ with a cavity size of 2 nm confines the bulk growth of metal NPs, resulting in ultra‐small (≈2 nm) and well‐dispersed metal NPs loaded in 2D C‐MOFs. The Pd or Pt NPs‐loaded Cu_3_(HHTP)_2_ exhibits remarkably improved NO_2_ sensing performance at room temperature due to the high reactivity of catalytic metal NPs and the high porosity of C‐MOFs. The catalytic effect of Pd and Pt NPs on NO_2_ sensing of Cu_3_(HHTP)_2_, in terms of reaction rate kinetics and activation energy, is demonstrated.

## Graphical abstract

**Ultra‐small and well‐dispersed metal nanoparticles (NPs)** are embedded in the cavities of 2D conductive metal–organic frameworks (C‐MOFs), for the synthesis of highly active and porous conductive materials. The catalytic metal NPs‐loaded C‐MOFs exhibit dramatically improved NO_2_ sensing performance at room temperature, due to the high reactivity of catalytic metal NPs and the high porosity of the electrically conductive MOFs.

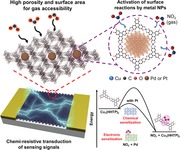

## 1 Introduction

Metal–organic frameworks (MOFs) have been extensively studied in various fields, such as gas separation,^1^, ^2^ gas storage,^3^, ^4^ catalysis,^5^, ^6^ energy storage system,^7^, ^8^ and sensors^9^, ^10^, ^11^ due to their ultrahigh porosity, large surface area, and tunable structures.^12^ In particular, MOFs are highly fascinating materials as gas‐sensing layers because the signals of gas sensors rely on surface reactions of analytes. However, most MOFs have low electrical conductivity due to the lack of the orbital overlap between metal nodes and organic ligands,^13^ impeding efficient signal transduction for gas sensing. Therefore, luminescence, localized surface plasmon resonance, interferometry, and electromechanical‐based MOF sensors have been introduced to transduce gas‐sensing signals,^9^, ^14^, ^15^ which complicates the sensor system.

Recently, conductive MOFs (C‐MOF) with high electrical conductivity and permanent porosity have been developed, exhibiting high feasibility for applications in supercapacitors,^16^, ^17^ electrocatalysts,^18^ and field‐effect transistors.^19^, ^20^ In addition, Dincă et al. first reported chemiresistive sensing properties of 2D C‐MOFs.^21^ They synthesized a conductive 2D Cu_3_(hexaiminotriphenylene)_2_, and demonstrated its impedance variations upon ammonia (NH_3_) adsorption. In addition, they developed the chemiresistive sensor arrays consisted of Cu and Ni‐based C‐MOFs sensing layers for the detection of diverse volatile organic compounds.^22^ Although C‐MOF‐based chemiresistors have been reported in a few articles, related with the detection of hydrogen sulfide (H_2_S), nitrogen monoxide (NO), and NH_3_ gases,^23^, ^24^, ^25^, ^26^ they still suffer from critical issues such as low sensitivity in air and poor cross‐sensitivity (selectivity), which hinders the practical application of C‐MOF‐based gas sensors. Besides, studies on C‐MOF‐based chemiresistive sensors are in the early stage, thus further in‐depth study is needed to address these limitations.

One of the noteworthy properties of MOFs is that their cavities can encapsulate noble metal nanoparticles (NPs), such as Au, Pt, and Pd.^27^ The unique porous structures of MOFs allow the encapsulation of ultra‐small and well‐dispersed metal NPs in their cavities. Since the reactivity and selectivity of catalysts highly rely on their surface properties,^28^ metal nanocatalysts embedded in highly porous MOFs are able to improve catalytic performance dramatically. For instance, Huo et al.^29^ synthesized Au or Pt NPs‐loaded zeolite imidazole frameworks (ZIF‐8) by using polyvinylpyrrolidone as a surfactant, which exhibited active and selective catalytic properties for hydrogenation. They further demonstrated a general and versatile synthesis of metal NPs‐loaded MOFs by extending the concept to various kinds of metals and MOFs.^30^ In addition, Yaghi et al.^31^ reported the site selective decoration of metal NPs in MOF and demonstrated their efficient catalytic performance in the conversion of methylcyclopentane. The functionalization of MOFs with metal NPs has been extensively studied to date.^32^, ^33^, ^34^ However, to the best of our knowledge, C‐MOFs decorated with metal NPs have not been reported.

Here, we propose metal NPs embedded in C‐MOFs, with integrated functionalities including moderate electrical conductivity and high porosity of C‐MOFs, and the outstanding catalytic reactivity of metal NPs. The unique porous structure of C‐MOFs inhibits the growth of metal NPs, thus ultra‐small and well‐dispersed metal NPs are embedded in the cavities of C‐MOFs. C‐MOFs provide numerous pores and high surface area, which are essential for the enhancement of the surface reaction, and their electrical conductivity realizes the direct transduction of signals from the surface reactions even at room temperature. The metal NPs in C‐MOFs not only promote the surface reactions but also tune the electrical properties of C‐MOFs, thereby improving the reactivity of C‐MOFs. As a proof of concept, we synthesized Pd or Pt NPs‐loaded 2D C‐MOFs that assembled by Cu nodes and 2,3,6,7,10,11‐hexahydroxytriphenylene (HHTP) linkers, for developing C‐MOFs‐based chemiresistors. Pd or Pt NPs (≈2 nm) functionalized Cu_3_(HHTP)_2_ (M@Cu_3_(HHTP)_2_, M = Pd or Pt) exhibited dramatically enhanced NO_2_ response, superior cross‐selectivity, and improved reaction kinetics (response speed). The important roles of metal NPs in C‐MOFs for the improvement of sensing characteristics are discussed based on the interpretation of the reaction kinetics and activation energy for NO_2_ adsorption.

## 2 Results and Discussion

**Figure 1:**
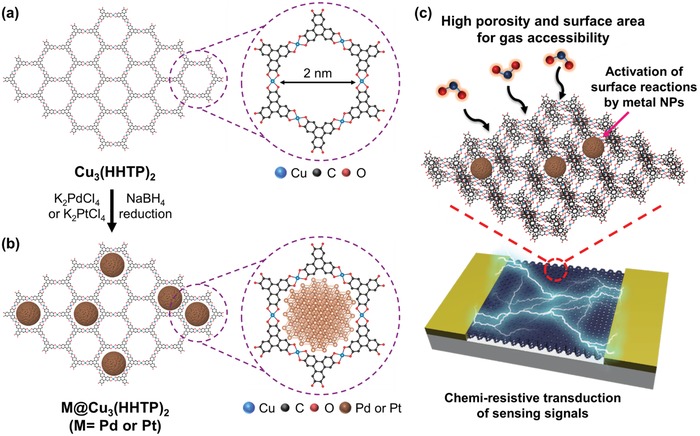
Schematic illustration of the synthesis of M@Cu_3_(HHTP)_2_. a) Cu_3_(HHTP)_2_ prepared by solvothermal synthesis and b) M@Cu_3_(HHTP)_2_ synthesized by the infiltration of metal ions and subsequent reduction process. c) The conceptual design of the M@Cu_3_(HHTP)_2_‐based gas sensors.

**Figure** **1** shows the synthetic process and conceptual design of metal NPs‐loaded Cu_3_(HHTP)_2_. First, Cu_3_(HHTP)_2_ was prepared by a solvothermal reaction of copper(II) acetate and HHTP in methanol solution. Cu_3_(HHTP)_2_ is an electrically conductive 2D extended framework (Figure 1a). After purification, Cu_3_(HHTP)_2_ was dispersed in deionized (DI) water, and metal salts were added into the suspension. To synthesize Pd or Pt NPs, potassium tetrachloroplatinate(II) and potassium tetrachloropalladate(II) were used as precursors, respectively. The metal ions are bound to oxygen sites in Cu_3_(HHTP)_2_.^35^ Then, the reduction by sodium borohydride (NaBH_4_) solution results in Pd or Pt NPs embedded in Cu_3_(HHTP)_2_ (Figure 1b). The porous structure of Cu_3_(HHTP)_2_ that consisted of permanent pores with a diameter of 2 nm, limits the growth of Pd or Pt, thereby creating ultra‐small and well‐dispersed metal NPs throughout the structure of 2D Cu_3_(HHTP)_2_. Then, we fabricated M@Cu_3_(HHTP)_2_‐based sensors to take advantages of fascinating features of M@Cu_3_(HHTP)_2_ (Figure 1c).

**Figure 2:**
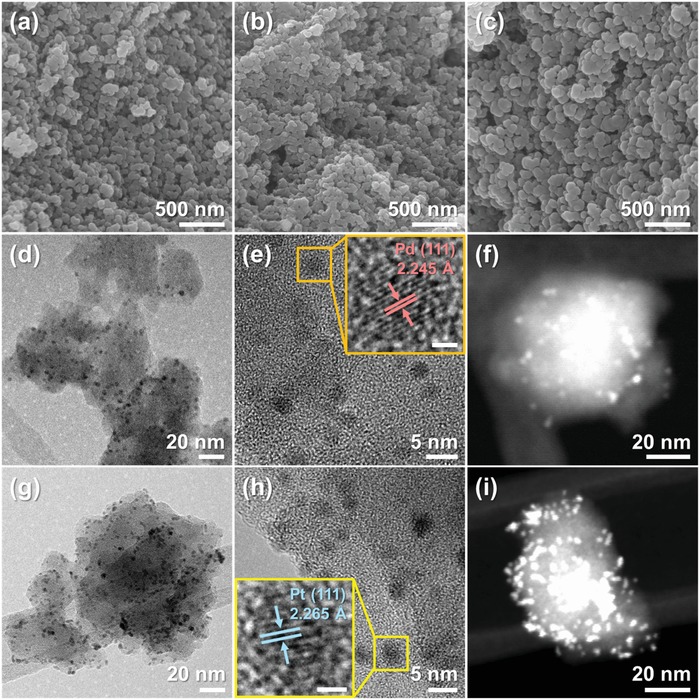
SEM images of a) pristine Cu_3_(HHTP)_2_, b) Pd@Cu_3_(HHTP)_2_, and c) Pt@Cu_3_(HHTP)_2_. d) TEM image of Pd@Cu_3_(HHTP)_2_, e) HRTEM image of Pd@Cu_3_(HHTP)_2_, and f) STEM image of Pd@Cu_3_(HHTP)_2_. g) TEM image of Pt@Cu_3_(HHTP)_2_, h) HRTEM image of Pt@Cu_3_(HHTP)_2_, and i) STEM image of Pt@Cu_3_(HHTP)_2_. The scale bars of the magnified images in (e) and (h) are 1 nm.

The scanning electron microscopy (SEM) image exhibited the particle‐shaped pristine Cu_3_(HHTP)_2_ with an average diameter of 40 nm (**Figure** **2**a). The morphologies of Pd@Cu_3_(HHTP)_2_ and Pt@Cu_3_(HHTP)_2_ were similar with that of pristine Cu_3_(HHTP)_2_ (Figure 2b,c). The transmission electron microscopy (TEM) image of Pd@Cu_3_(HHTP)_2_ clearly showed that ultra‐small Pd NPs were well‐dispersed in Cu_3_(HHTP)_2_ (Figure 2d). The high‐resolution TEM (HRTEM) image of Cu_3_(HHTP)_2_ revealed the lattice distance of 2.245 Å (Figure 2e), which is corresponded to the crystal plane of Pd (111). In addition, the size of Pd NPs was identified to be ≈2 nm, demonstrating the confined growth of Pd NPs in the cavities of Cu_3_(HHTP)_2_. The dark‐field scanning TEM (STEM) analysis also confirmed that the nanoscale Pd was well‐dispersed in Cu_3_(HHTP)_2_ (Figure 2f). The energy‐dispersive X‐ray spectroscopy (EDS) elemental mapping images of Pd@Cu_3_(HHTP)_2_ showed C, Cu, O, and Pd elements (Figure S1a, Supporting Information). However, the intensity of Pd was relatively low. In the case of Pt@Cu_3_(HHTP)_2_, the samples showed a similar microstructure with that of Pd@Cu_3_(HHTP)_2_. As shown in Figure 2g, the tiny‐sized Cu_3_(HHTP)_2_ was decorated by Pt NPs with an average diameter of 2 nm, which were also well‐dispersed in Cu_3_(HHTP)_2_. The lattice fringe of Pt (111) plane with the spacing of 2.265 Å was clearly observed in the HRTEM image (Figure 2h). In addition, the STEM image of Pt@Cu_3_(HHTP)_2_ revealed that Pt NPs are well dispersed throughout the structure of the Cu_3_(HHTP)_2_ (Figure 2i). The presence of C, Cu, O, and Pt elements in the samples was confirmed by EDS elemental mapping (Figure S1b, Supporting Information). These results demonstrated the versatile synthesis of the metal NPs‐loaded C‐MOFs. Therefore, the proposed synthesis can be easily extended to various types of metal NPs‐loaded C‐MOFs by varying the kind of metals and C‐MOFs for the development of highly active and conductive porous materials.

**Figure 3:**
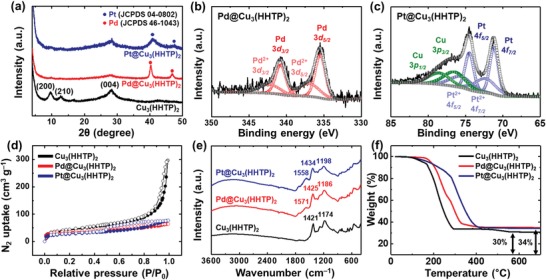
a) XRD analysis of Cu_3_(HHTP)_2_, Pd@Cu_3_(HHTP)_2_, and Pt@Cu_3_(HHTP)_2_. XPS spectra of b) Pd@Cu_3_(HHTP)_2_ for Pd 3d and c) Pt@Cu_3_(HHTP)_2_ for Pt 4f. d) N_2_ adsorption and desorption isotherms of the samples at 77 K. e) FT‐IR spectra of Cu_3_(HHTP)_2_, Pd@Cu_3_(HHTP)_2_, and Pt@Cu_3_(HHTP)_2_. f) TGA of the samples under air atmosphere.

The crystal structures of pristine Cu_3_(HHTP)_2_, Pd@Cu_3_(HHTP)_2_, and Pt@Cu_3_(HHTP)_2_ were investigated by X‐ray diffraction (XRD) analysis (**Figure** **3**a). The pristine Cu_3_(HHTP)_2_ exhibited the crystal planes of (200), (210), and (004), which were similar to observations in previous literature.^36^ After the functionalization of Cu_3_(HHTP)_2_ with metal NPs, the peaks related to Pd and Pt were clearly observed in the XRD patterns of Pd@Cu_3_(HHTP)_2_ and Pt@Cu_3_(HHTP)_2_ (red dots for *fcc* Pd (JCPDS no. 46‐1043) and blue dots for *fcc* Pt (JCPDS no. 04‐0802) in Figure 3a), while the Cu_3_(HHTP)_2_ peaks were weak due to their low intensity. The chemical state of Pd and Pt in Cu_3_(HHTP)_2_ were verified by X‐ray photoelectron spectrometer (XPS) analysis. The XPS survey spectra and the high‐resolution spectra of Pd@Cu_3_(HHTP)_2_ and Pt@Cu_3_(HHTP)_2_ revealed the presence of Cu, O, and C elements (Figures S2 and S3, Supporting Information). In the case of Pd@Cu_3_(HHTP)_2_, the Pd 3d peaks in the XPS spectrum revealed the two characteristics peaks at 335.5 eV for Pd^0^ 3d_5/2_ and 336.9 eV for Pd^2+^ 3d_5/2_ with an energy gap of 5.3 eV between 3d_5/2_ and 3d_3/2_ peaks (Figure 3b).^37^ On the other hand, the high‐resolution spectrum of Pt@Cu_3_(HHTP)_2_ in the vicinity of Pt 4f was deconvoluted into Pt^0^ at 71.2 eV and Pt^2+^ at 72.4 eV for 4f_7/2_, with background peaks related to Cu 3p, which correspond to metallic Pt and PtO, respectively (Figure 3c).^38^, ^39^ The XPS analysis of the samples confirmed that the metallic Pd and Pt NPs were embedded in the Cu_3_(HHTP)_2_ and that some of the metal NPs were partially oxidized to PdO and PtO.

The N_2_ adsorption and desorption isotherms at 77 K revealed the porous structure of the samples (Figure 3d). The N_2_ uptake of pristine Cu_3_(HHTP)_2_, Pd@Cu_3_(HHTP)_2_, and Pt@Cu_3_(HHTP)_2_ rapidly increased at low relative pressure (type I isotherms), revealing that the microporous structure of pristine Cu_3_(HHTP)_2_ was well preserved after the metal encapsulation. The increased N_2_ uptake of pristine Cu_3_(HHTP)_2_ at high relative pressure can be described by the pores between agglomerated Cu_3_(HHTP)_2_ particles, while this sharp increase did not appear after metal encapsulation. The specific surface areas calculated by Brunauer–Emmett–Teller (BET) method were 151.95 m^2^ g^−1^ for pristine Cu_3_(HHTP)_2_, 122.32 m^2^ g^−1^ for Pd@Cu_3_(HHTP)_2_, and 97.35 m^2^ g^−1^ for Pt@Cu_3_(HHTP)_2_, respectively. The decrease in the surface area was attributed to the mass contribution of nonporous metal NPs in M@Cu_3_(HHTP)_2_. To further investigate the structural stability of the samples, we carried out Fourier transform infrared (FT‐IR) analysis. The FT‐IR spectrum of pristine Cu_3_(HHTP)_2_ exhibited the major peaks at 1174 cm^−1^ for C–O stretching vibration and 1421 cm^−1^ for C–H scissoring vibration (Figure 3e).^40^ The Pd@Cu_3_(HHTP)_2_ and Pt@Cu_3_(HHTP)_2_ showed FT‐IR patterns similar to that of pristine Cu_3_(HHTP)_2_, indicating that the structure of Cu_3_(HHTP)_2_ was retained after the loading of Pd and Pt NPs. The new peaks at 1571 cm^−1^ for Pd@Cu_3_(HHTP)_2_ and at 1558 cm^−1^ for Pt@Cu_3_(HHTP)_2_ were associated with the vibration of adsorbed H_2_O on metal NPs,^41^ revealing the presence of metal NPs in the M@Cu_3_(HHTP)_2_. The loading amounts of Pd and Pt in Cu_3_(HHTP)_2_ were analyzed by using inductively coupled plasma optical emission spectrometry. The relative weight ratio of Pd and Pt was verified to be 3.3 wt% in Pd@Cu_3_(HHTP)_2_ and 5.6 wt% in Pt@Cu_3_(HHTP)_2_, respectively. The thermal gravimetric analysis (TGA) further confirmed the loading amounts of Pd and Pt (about 4 wt% in both samples), which remained as residues upon decomposition of organic matter at high temperature (Figure 3f). In addition, we observed that the thermal stability of Cu_3_(HHTP)_2_ was slightly improved after the loading of metal NPs, which perhaps resulted from the interaction of Pd and Pt with Cu_3_(HHTP)_2_.

To verify the high reactivity of metal NPs in C‐MOFs, we conducted gas‐sensing measurements against nitrogen dioxide (NO_2_) molecules by using Cu_3_(HHTP)_2_, Pd@Cu_3_(HHTP)_2_, and Pt@Cu_3_(HHTP)_2_. NO_2_ is one of the toxic gases, and can be emitted from industrial sources such as vehicles and power plants, raising serious concerns on environment and human health.^42^, ^43^ Thus, it is important to develop highly sensitive sensors capable of detecting NO_2_ in the order of sub‐part per million (ppm) levels. The sensors were fabricated by the drop‐coating of the samples on an alumina (Al_2_O_3_) substrate patterned with two parallel Au electrodes. The sensing characteristics were evaluated at room temperature in dry air (relative humidity: ≈5%). It was noted that the baseline resistance of the sensors in humid atmospheres (relative humidity: ≈95%) continuously increased to the measurement limit (100 MΩ) of our sensing system. This is because H_2_O molecules (hundreds to tens of thousands of ppm levels) in air can be easily adsorbed on the open Cu sites in Cu_3_(HHTP)_2_.^44^ The baseline resistances of the samples in dry air were 13 MΩ for pristine Cu_3_(HHTP)_2_, 25 MΩ for Pd@Cu_3_(HHTP)_2_, and 63 MΩ for Pt@Cu_3_(HHTP)_2_ (Figure S4, Supporting Information). The increase in baseline resistances was attributed to the creation of multiple junctions between materials of different work functions (5.99 eV for Cu_3_(HHTP)_2_, 5.12 eV for Pd, and 5.65 eV for Pt).^26^, ^45^ Because the majority current carriers in Cu_3_(HHTP)_2_ are holes,^26^ the electrons transfer from Pd or Pt to Cu_3_(HHTP)_2_ increase the baseline resistance of the sensors. In addition, the refinement of XRD data revealed that the interlayer spacing (*z*‐axis) of Pd@Cu_3_(HHTP)_2_ and Pt@Cu_3_(HHTP)_2_ slightly increased (3.1531 Å for Pd@Cu_3_(HHTP)_2_ and 3.1543 Å for Pt@Cu_3_(HHTP)_2_) compared with that (3.1518 Å) of pristine Cu_3_(HHTP)_2_ (Table S1, Supporting Information). The slight increase of lattice spacing to *z*‐axis can reduce the hopping current between C‐MOF layers.^44^ Moreover, the partially oxidized Pd and Pt (PdO and PtO as confirmed with XPS analysis) can further affect the baseline resistance by creating additional junctions. Since the work functions of PdO and PtO_*x*_ are 7.90 and 5.65 eV, respectively,^46^, ^47^ PtO donates electrons to Cu_3_(HHTP)_2_, whereas PdO deprives Cu_3_(HHTP)_2_ of electrons (Figure S5, Supporting Information). Therefore, from these reasons, the baseline resistance of Pt@Cu_3_(HHTP)_2_ was much higher than that of Pd@Cu_3_(HHTP)_2_.

**Figure 4:**
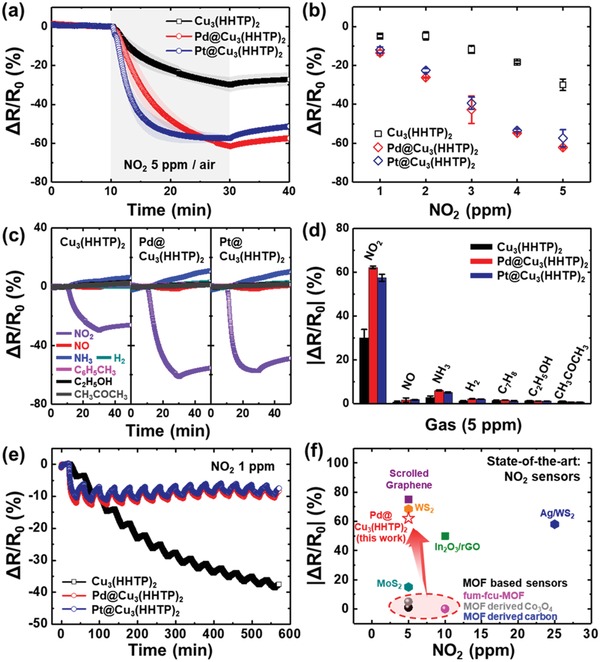
Sensing characteristics of Cu_3_(HHTP)_2_, Pd@Cu_3_(HHTP)_2_, and Pt@Cu_3_(HHTP)_2_ operated at room temperature in air. a) Dynamic response variations of the sensors to 5 ppm of NO_2_, b) response versus NO_2_ concentrations, c) dynamic response changes of the sensors when exposed to 5 ppm of diverse gas species (NO, NH_3_, H_2_, C_7_H_8_, C_2_H_5_OH, and CH_3_COCH_3_), d) calculated response toward various analytes, and e) response variations of the sensors during cyclic sensing tests toward 1 ppm of NO_2_. f) Response versus NO_2_ concentration for diverse state‐of‐the‐art NO_2_ sensors operated at room temperature in air.

The variations in the sensor resistances upon exposure to 5 ppm of NO_2_ were monitored in real time at room temperature in air (Figure S4, Supporting Information). The dynamic resistance variations were normalized to dynamic response transitions. The response is defined by the ratio of the resistance change to the baseline resistance (Δ*R*/*R*_0_) (**Figure** **4**a). The sensors showed slow recovery speed, due to the difficulty in desorption of NO_2_ molecules at room temperature. Because NO_2_ is a strong electron acceptor, the resistance of the Cu_3_(HHTP)_2_, which exhibits a p‐type semiconducting behavior,^26^ decreased by the adsorption of NO_2_. When Pd@Cu_3_(HHTP)_2_ and Pt@Cu_3_(HHTP)_2_ were exposed to NO_2_, their resistances drastically decreased compared with that of pristine Cu_3_(HHTP)_2_. The Pd@Cu_3_(HHTP)_2_ and Pt@Cu_3_(HHTP)_2_ showed much improved response to 5 ppm of NO_2_ (−62.11% for Pd@Cu_3_(HHTP)_2_ and −57.38% for Pt@Cu_3_(HHTP)_2_) than pristine Cu_3_(HHTP)_2_ (−29.95%). In addition, Pd@Cu_3_(HHTP)_2_ and Pt@Cu_3_(HHTP)_2_ exhibited higher response to 1 ppm of NO_2_ (−13.5% for Pd@Cu_3_(HHTP)_2_ and −12.1% for Pt@Cu_3_(HHTP)_2_)) than pristine Cu_3_(HHTP)_2_ (−5.0%) (Figure 4b and Figure S6, Supporting Information). Response time of Cu_3_(HHTP)_2_ was also hugely improved by embedding Pd or Pt NPs in Cu_3_(HHTP)_2_ (Figure S7, Supporting Information). In particular, the response time to 1 ppm of NO_2_ was 13.8 min for Pd@Cu_3_(HHTP)_2_, 14 min for Pt@Cu_3_(HHTP)_2_, and 18 min for Cu_3_(HHTP)_2_. This result indicates that the M@Cu_3_(HHTP)_2_‐based sensors can detect 1 ppm of NO_2_ molecules within 15 min, which is the short‐term permissible exposure limit designated by the Occupational Safety and Health Administration (OSHA) in the United States.^48^ These results confirm that the sensing properties are significantly improved after the decoration Pd or Pt NPs on Cu_3_(HHTP)_2_.

Moreover, we examined the selectivity of Cu_3_(HHTP)_2_, Pd@Cu_3_(HHTP)_2_, and Pt@Cu_3_(HHTP)_2_ sensing layers. The Pd@Cu_3_(HHTP)_2_ and Pt@Cu_3_(HHTP)_2_ exhibited high resistance changes when they were exposed to NO_2_, and negligible resistance changes upon exposure to nitrogen monoxide (NO), ammonia (NH_3_), hydrogen (H_2_), toluene (C_6_H_5_CH_3_), ethanol (C_2_H_5_OH), and acetone (CH_3_COCH_3_) (Figure 4c). The normalized response values of Pd@Cu_3_(HHTP)_2_ (62.11%) and Pt@Cu_3_(HHTP)_2_ (57.38%) toward NO_2_ molecules were tenfold higher than those (lower than 5.90% for Pd@Cu_3_(HHTP)_2_ and lower than 5.03% for Pt@Cu_3_(HHTP)_2_) toward interfering gases (Figure 4d), demonstrating the high NO_2_ selectivity of the catalyst‐loaded C‐MOFs sensors. In terms of repeatability, the resistance of pristine Cu_3_(HHTP)_2_ did not recover to the baseline value during 14 cyclic sensing tests toward 1 ppm of NO_2_. However, the Pd@Cu_3_(HHTP)_2_ and Pt@Cu_3_(HHTP)_2_ showed stable response and recovery dynamics during the cyclic tests (Figure 4e), demonstrating that the response and recovery kinetics of the sensors to low levels of NO_2_ were improved by the addition of Pd and Pt NPs. Note that the response of Pd@Cu_3_(HHTP)_2_ and Pt@Cu_3_(HHTP)_2_ slightly decreased as the cycling number increased, which was originated from the residual adsorbed NO_2_ that gives rise to the change of NO_2_ adsorption equilibrium.

The response values (Δ*R*/*R*_0_) of NO_2_ sensors reported in recent literatures are illustrated in Figure 4f, and the detailed sensing properties are summarized in Table S2 in the Supporting Information.^49^, ^50^, ^51^, ^52^, ^53^, ^54^, ^55^, ^56^ Although the response of Pd@Cu_3_(HHTP)_2_ was slightly lower than those of other materials, a significant improvement in MOF‐based sensors has been achieved in this work by combining the metal NPs with C‐MOFs. The Pd@Cu_3_(HHTP)_2_ exhibited the highest NO_2_ response among MOF‐based sensors reported to date, including MOFs, C‐MOFs, and MOF derivatives. The response of Pd@Cu_3_(HHTP)_2_ increased by more than 12 times with improved reaction kinetics even at room temperature, compared with the response of reported MOFs‐based sensors. These outstanding results confirmed the superiority of metal NPs‐loaded C‐MOFs, as a highly active material with electrical conductivity, for applications in NO_2_ sensors operated at room temperature. Although the stability of M@Cu_3_(HHTP)_2_ against humidity was not demonstrated here due to easy adsorption of H_2_O on Cu_3_(HHTP)_2_ at high humidity, we suppose that the introduction of additional catalysts or humidity screening membranes, as previously demonstrated,^57^, ^58^ can improve the sensing properties even in humid atmospheres.

**Figure 5:**
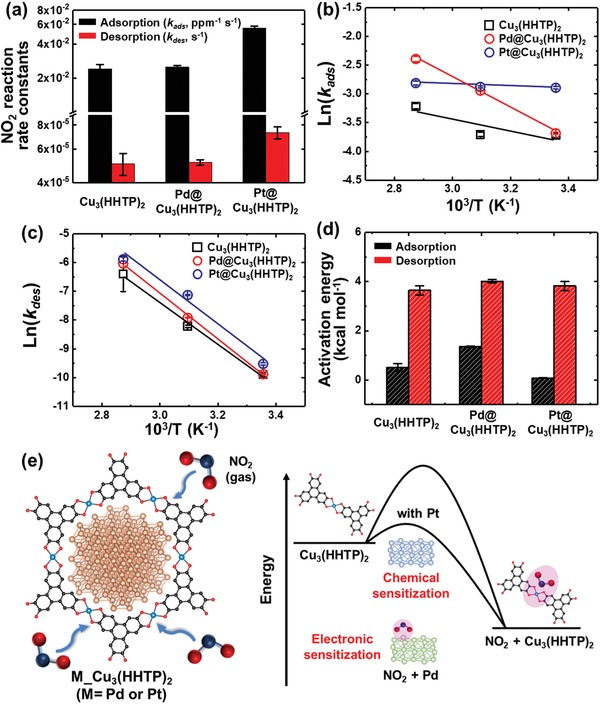
a) Calculated adsorption rate constants (*k*_ads_) and desorption rate constants (*k*_des_) of the sensors. Arrhenius plots for b) the adsorption rate constants and c) the desorption rate constants. d) Calculated activation energy for the adsorption and desorption of NO_2_ on the sensors. e) Schematic illustration of sensing mechanism of the sensors.

In general, the adsorption of NO_2_ deprived sensing materials of electrons, thereby changing the resistance of the sensors.^59^, ^60^ In the case of C‐MOFs, the chemiresistive sensing behaviors originate from the charge transfer between C‐MOFs and adsorbed gas molecules.^22^ Analytes are preferentially adsorbed on open metal sites that are coordinatively unsaturated metal centers in MOFs, changing their electrical resistance.^44^ Since NO_2_ is an electron acceptor at room temperature, the resistance of Cu_3_(HHTP)_2_, a p‐type sensing material, decreases when C‐MOFs were exposed to NO_2_. The highly porous Cu_3_(HHTP)_2_ with MΩ‐level resistance and high surface area not only facilitate gas diffusion into sensing layers through a number of pores but also transduce electrical signals from the surface reaction. In addition, the metal NPs embedded in the cavities of C‐MOFs can activate the surface reaction, enabling effective modulation of resistance upon exposure to target analytes. To investigate the catalytic effect of Pd and Pt NPs on the NO_2_ sensing behavior of Cu_3_(HHTP)_2_, we calculated NO_2_ response and recovery kinetics of the sensors based on i) the mass action law of NO_2_ adsorption reaction on sensing materials (Cu_3_(HHTP)_2_ or M@Cu_3_(HHTP)_2_) (Reactions 1 and 2) and ii) the assumption that response is proportional to the amounts of gas adsorbed.^60^ The adsorption rate constant (*k*_ads_), desorption rate constant (*k*_des_), and equilibrium constant (*K* = *k*_ads_/*k*_des_) were obtained from the first reaction of the sensors to 5 ppm of NO_2_ by the exponential fitting of the response versus time curves (*R*(*t*)) (Figure S8, Supporting Information) as shown in the equations below

(1) $${\mathrm{NO}}_{2}\left(\mathrm{gas}\right)+{\mathrm{Cu}}_{3}{\left(\mathrm{HHTP}\right)}_{2}\left(s\right)↔{\mathrm{NO}}_{2}-\;{\mathrm{Cu}}_{3}{\left(\mathrm{HHTP}\right)}_{2}\left(s\right)$$

(2) $${\mathrm{NO}}_{2}\left(\mathrm{gas}\right)+M@{\mathrm{Cu}}_{3}{\left(\mathrm{HHTP}\right)}_{2}\left(s\right)↔{\mathrm{NO}}_{2}-M@{\mathrm{Cu}}_{3}{\left(\mathrm{HHTP}\right)}_{2}\left(s\right)$$

(3) $$R\left(t\right)\;\mathrm{for}\;{\mathrm{NO}}_{2}\;\mathrm{adsorption}\;=R_{\max}\cdot \frac{C_{a}K}{1+C_{a}K}\left(1-\exp\left[-\frac{1+C_{g}K}{K}\cdot k_{\mathrm{ads}}\cdot t\right]\right)$$

(4) $$R\left(t\right)\;\mathrm{for}\;{\mathrm{NO}}_{2}\;\mathrm{desorption}\;=R_{0}\;\exp\left[-k_{\mathrm{des}}\cdot t\right]$$

where *R*_max_ is the maximum response of the sensors, *C*_a_ is the concentration of analytes (NO_2_), and *R*_0_ is the response of the sensors before refreshing with air. The calculated adsorption and desorption rate constants are described in **Figure** **5**a. The Pt@Cu_3_(HHTP)_2_ exhibited higher NO_2_ adsorption and desorption kinetics (5.54 × 10^−2^ ppm^−1^ s^−1^ for adsorption and 7.30 × 10^−5^ s^−1^ for desorption) than pristine Cu_3_(HHTP)_2_ (2.43 × 10^−2^ ppm^−1^ s^−1^ for adsorption and 5.03 × 10^−5^ s^−1^ for desorption), demonstrating the activation of NO_2_ reaction by Pt NPs. On the other hand, Pd@Cu_3_(HHTP)_2_ showed a slightly improved NO_2_ reaction rate kinetics (2.51 × 10^−2^ ppm^−1^ s^−1^ for adsorption and 5.10 × 10^−5^ s^−1^ for desorption) compared with pristine Cu_3_(HHTP)_2_. To further understand the catalytic effect of Pd and Pt NPs, we investigated the activation energy (*E*_a_) of the sensors for NO_2_ adsorption and desorption by using Arrhenius equation

(5) $$\mathrm{Reaction}\;\mathrm{rate}\;\mathrm{constant}\;\left(k_{\mathrm{ads}}\;or\;k_{\mathrm{des}}\right)\;=A_{0}\;\exp\left[-E_{a}/RT\right]$$

where *A*_0_ is a pre‐exponential factor, *E*_a_ is the activation energy for the reaction, and *R* is the universal gas constant. Considering that Cu_3_(HHTP)_2_ is not stable over 100 °C in air (Figure 3f), we conducted additional sensing measurements using 5 ppm of NO_2_ at 50 and 75 °C to investigate the variation of reaction rate constants at different operating temperatures. Then, we calculated the NO_2_ adsorption and desorption rate constants of the samples by using Equations (1) and (2) (Figures S9 and S10, Supporting Information). As the operating temperature increased, the adsorption and desorption rate constants also increased due to the high thermal energy of analytes (Table S3, Supporting Information). The activation energy of the samples was calculated from the slope (−*E*_a_/*R*) of the natural logarithm of the reaction rate constants (*k*_ads_ or *k*_des_) versus the inverse of the operating temperature (1/*T*) (Figure 5b,c). The calculated activation energy for NO_2_ adsorption was 511.5 cal mol^−1^ for pristine Cu_3_(HHTP)_2_, 1351.5 cal mol^−1^ for Pd@Cu_3_(HHTP)_2_, and 83.4 cal mol^−1^ for Pt@Cu_3_(HHTP)_2_, whereas that for NO_2_ desorption was 3644.8 cal mol^−1^ for pristine Cu_3_(HHTP)_2_, 4015.7 cal mol^−1^ for Pd@Cu_3_(HHTP)_2_, and 3822.7 cal mol^−1^ for Pt@Cu_3_(HHTP)_2_ (Figure 5d). The activation energy of NO_2_ adsorption dramatically decreased with loading of Pt NPs, indicating the chemical catalytic effect of Pt NPs that facilitate NO_2_ adsorption on Cu_3_(HHTP)_2_ (Figure 5e). The NO_2_ spill‐over was also observed in the system of Pt NPs with supporting materials (NO_2_ adsorbents).^61^ On the other hand, the decoration of Pd NPs increased the activation energy of NO_2_ adsorption and desorption compared with pristine Cu_3_(HHTP)_2_, revealing that the resistance change of Pd@Cu_3_(HHTP)_2_ during NO_2_ adsorption is not solely related to the charge transfers between NO_2_ and Cu_3_(HHTP)_2_. It is reported that NO_2_ molecules are adsorbed on Pd NPs in the form of nitrito geometry.^62^ The NO_2_ adsorption on Pd NPs withdrew electrons from Pd, lowering the potential barriers of Schottky junctions between Pd NPs and Cu_3_(HHTP)_2_. Therefore, the resistance of the sensors was decreased by the electronic catalytic effect of Pd NPs upon exposure to NO_2_. This mechanism of the electronic sensitization is also worked for the Pt catalysts on Cu_3_(HHTP)_2_ because NO_2_ can be adsorbed on Pt NPs. Although the minor phases of oxidized metal NPs (PdO and PtO NPs) can differently affect the sensing properties, the calculation from the sensing results included the effect of both the major phases of metallic Pd and Pt and the minor phases of PdO and PtO in Cu_3_(HHTP)_2_‐based NO_2_ sensors. Therefore, it is apparent that the sensing properties of Cu_3_(HHTP)_2_ were dramatically enhanced by the catalytic effect of Pd and Pt NPs in Cu_3_(HHTP)_2_.

## 3 Conclusion

In conclusion, we confirmed the facile synthesis of Pd and Pt NPs‐loaded Cu_3_(HHTP)_2_ and their potential applications in NO_2_ sensors. The porous structure of C‐MOFs effectively limits the growth of Pd and Pt NPs, thereby creating ultra‐small (≈2 nm) and well‐dispersed catalyst NPs in their cavities. The chemical and electronic catalytic effect of Pd and Pt NPs dramatically improved the NO_2_ sensing properties of Cu_3_(HHTP)_2_‐based sensors in terms of response, selectivity, and enhanced response and recovery speed. These results demonstrated the high feasibility of Pd@Cu_3_(HHTP)_2_ and Pt@Cu_3_(HHTP)_2_ as gas‐sensing layers for room temperature NO_2_ detection. To the best of our knowledge, this is first report on metal NPs embedded in C‐MOFs as highly active and conductive porous sensing materials. Considering that the synthetic versatility of C‐MOFs has been established, various combinations of metal NPs and C‐MOFs can be easily explored, providing new materials platform for diverse applications.

## 4 Experimental Section

*Materials*: 2,3,6,7,10,11‐hexahydroxytriphenylene hydrate (HHTP, C_18_H_12_O_6_·H_2_O, 95.0%) was purchased from Tokyo Chemical Industry. Copper(II) acetate monohydrate (Cu(CO_2_CH_3_)·H_2_O, 99.9%), methanol (CH_3_OH, 99.9%), ethanol (C_2_H_5_OH 99.5%), and acetone (CH_3_COCH_3_, 99.5%) were purchased from Sigma‐Aldrich. Potassium tetrachloroplatinate(II) (K_2_PtCl_4_), potassium tetrachloropalladate(II) (K_2_PdCl_4_), and sodium borohydride (NaBH_4_, 96%) were purchased from Aldrich. All materials were used as received.

*Synthesis of Cu_3_(HHTP)_2_*: Cu_3_(HHTP)_2_ was prepared by solvothermal synthesis. 0.320 g of copper(II) acetate monohydrate was dissolved in 100 mL of methanol, whereas 0.260 g of HHTP was separately dissolved in 125 mL of methanol. Two solutions were mixed in a 500 mL capped bottle, and vigorously stirred by using a magnetic bar at room temperature for 10 min. Then, the mixture was heated in a box furnace at 65 °C for 24 h, resulting in dark solutions. The solution was naturally cooled to room temperature, and the upper transparent solvent was removed. The samples were washed with fresh methanol (three times) and acetone (two times) by centrifugation, and dried at 50 °C for 12 h in air and at 100 °C for 12 h in vacuum.

*Synthesis of M@Cu_3_(HHTP)_2_ (M = Pd or Pt)*: M@Cu_3_(HHTP)_2_ was prepared by the infiltration of metal ions followed by a reduction process. 40 mg of Cu_3_(HHTP)_2_ was homogenously dispersed in 5 mL of DI water. 5 mg of potassium tetrachloroplatinate(II) or potassium tetrachloropalladate(II) was added into the suspension, and the mixture was stirred for 30 min. To reduce metal ions in the cavities of Cu_3_(HHTP)_2_, NaBH_4_ solution (1 mg mL^−1^) was added to the mixture. After 30 min, the samples were purified by centrifugation and washing with DI water (three times) and acetone (two times). Lastly, for activation, the samples were dried at 50 °C for 6 h in air and at 100 °C for 12 h in vacuum.

*Material Characterization*: SEM (XL30, Philips) and TEM (Tecnai G2 F30 S‐Twin, FEI) were used to investigate the morphology and microstructure of the samples. The crystal structure was investigated by XRD (SmartLab, Rigaku) analysis with Cu Kα radiation (λ = 1.5418 Å). The composition and chemical binding states of the samples were investigated by XPS (Sigma Probe, Thermo VG Scientific). N_2_ adsorption/desorption isotherms at 77 K (Tristar 3020, Micromeritics) were carried out to investigate the porous structure of the samples. The surface area was calculated from the N_2_ isotherms of the samples by using BET method.

*Gas‐Sensing Measurement*: 6 mg of Cu_3_(HHTP)_2_, Pd@Cu_3_(HHTP)_2_, and Pt@Cu_3_(HHTP)_2_ were independently dispersed in 300 mL of ethanol, and sonicated for 10 min. Then, the dispersion solution of each sensing material was drop‐coated on the alumina (Al_2_O_3_) substrate (dimension: 2.5 mm × 2.5 mm and thickness: 0.2 mm). The alumina substrate was patterned with two parallel Au electrodes (width: 25 µm, distance: 70 µm) on the top side and a Pt microheater on its back side. 5 µL of the suspension was drop‐coated on the sensing substrate three times. The sensing measurements were carried out in a sealed chamber at room temperature and dry condition (relative humidity = 5%). To control the operating temperature (50 and 75 °C), a voltage was applied to the Pt microheater using a DC power supply (E3647A, Agilent). The sensors were stabilized in air for 3 h before each sensing test. Then, the sensors were exposed to target gas, and subsequently recovered by air injection. To investigate the sensing properties of the sensors, nitrogen dioxide (NO_2_), ammonia (NH_3_), hydrogen (H_2_), toluene (C_6_H_5_CH_3_), ethanol (C_2_H_5_OH), and acetone (CH_3_COCH_3_) were injected into the chamber, in turn. The dynamic resistance of the sensors was obtained in real time by using a data acquisition system (34972, Agilent). The response was calculated as the ratio of the sensor resistance before and after exposure to analytes [(Δ*R*/*R*_0_) × 100], (%)], where Δ*R* is the resistance variation of the sensors when exposed to gas and *R*_0_ is the baseline resistance of the sensors when exposed to air. The error bars were added by multiple sensing tests of three independent sensors for each sensing material.

## Conflict of Interest

The authors declare no conflict of interest.

## Supporting information

Supplementary

## References

[1] J.‐R. Li , R. J. Kuppler , H.‐C. Zhou , Chem. Soc. Rev. 2009, 38, 1477. 19384449 10.1039/b802426j

[2] G. Liu , V. Chernikova , Y. Liu , K. Zhang , Y. Belmabkhout , O. Shekhah , C. Zhang , S. Yi , M. Eddaoudi , W. J. Koros , Nat. Mater. 2018, 17, 283. 29434309 10.1038/s41563-017-0013-1

[3] Y. Lin , C. Kong , Q. Zhang , L. Chen , Adv. Energy Mater. 2017, 7, 1601296.

[4] J.‐R. Li , Y. Ma , M. C. McCarthy , J. Sculley , J. Yu , H.‐K. Jeong , P. B. Balbuena , H.‐C. Zhou , Coord. Chem. Rev. 2011, 255, 1791.

[5] J. Lee , O. K. Farha , J. Roberts , K. A. Scheidt , S. T. Nguyen , J. T. Hupp , Chem. Soc. Rev. 2009, 38, 1450. 19384447 10.1039/b807080f

[6] J. Liang , Z. Liang , R. Zou , Y. Zhao , Adv. Mater. 2017, 29, 1701139. 10.1002/adma.201701139 28628246

[7] W. Xia , A. Mahmood , R. Zou , Q. Xu , Energy Environ. Sci. 2015, 8, 1837.

[8] H. Wang , Q.‐L. Zhu , R. Zou , Q. Xu , Chem 2017, 2, 52.

[9] L. E. Kreno , K. Leong , O. K. Farha , M. Allendorf , R. P. Van Duyne , J. T. Hupp , Chem. Rev. 2012, 112, 1105. 22070233 10.1021/cr200324t

[10] W.‐T. Koo , S. Qiao , A. F. Ogata , G. Jha , J.‐S. Jang , V. T. Chen , I.‐D. Kim , R. M. Penner , ACS Nano 2017, 11, 9276. 28820935 10.1021/acsnano.7b04529

[11] W.‐T. Koo , S.‐J. Choi , S.‐J. Kim , J.‐S. Jang , H. L. Tuller , I.‐D. Kim , J. Am. Chem. Soc. 2016, 138, 13431. 27643402 10.1021/jacs.6b09167

[12] H. Furukawa , K. E. Cordova , M. O'Keeffe , O. M. Yaghi , Science 2013, 341, 1230444. 23990564 10.1126/science.1230444

[13] L. Sun , M. G. Campbell , M. Dincă , Angew. Chem., Int. Ed. 2016, 55, 3566. 10.1002/anie.201506219 26749063

[14] S. Achmann , G. Hagen , J. Kita , I. M. Malkowsky , C. Kiener , R. Moos , Sensors 2009, 9, 1574. 22573973 10.3390/s90301574 PMC3345849

[15] Z. Hu , B. J. Deibert , J. Li , Chem. Soc. Rev. 2014, 43, 5815. 24577142 10.1039/c4cs00010b

[16] D. Feng , T. Lei , M. R. Lukatskaya , J. Park , Z. Huang , M. Lee , L. Shaw , S. Chen , A. A. Yakovenko , A. Kulkarni , J. Xiao , K. Fredrickson , J. B. Tok , X. Zou , Y. Cui , Z. Bao , Nat. Energy 2018, 3, 30.

[17] D. Sheberla , J. C. Bachman , J. S. Elias , C.‐J. Sun , Y. Shao‐Horn , M. Dincă , Nat. Mater. 2017, 16, 220. 27723738 10.1038/nmat4766

[18] E. M. Miner , T. Fukushima , D. Sheberla , L. Sun , Y. Surendranath , M. Dincă , Nat. Commun. 2016, 7, 10942. 26952523 10.1038/ncomms10942 PMC4786780

[19] A. A. Talin , A. Centrone , A. C. Ford , M. E. Foster , V. Stavila , P. Haney , R. A. Kinney , V. Szalai , F. El Gabaly , H. P. Yoon , F. Leonard , M. D. Allendorf , Science 2013, 343, 66. 24310609 10.1126/science.1246738

[20] G. Wu , J. Huang , Y. Zang , J. He , G. Xu , J. Am. Chem. Soc. 2017, 139, 1360. 27794592 10.1021/jacs.6b08511

[21] M. G. Campbell , D. Sheberla , S. F. Liu , T. M. Swager , M. Dincă , Angew. Chem., Int. Ed. 2015, 54, 4349. 10.1002/anie.201411854 25678397

[22] M. G. Campbell , S. F. Liu , T. M. Swager , M. Dincaă , J. Am. Chem. Soc. 2015, 137, 13780. 26456526 10.1021/jacs.5b09600

[23] M. G. Campbell , M. Dincă , Sensors 2017, 17, 1108.

[24] M. Ko , A. Aykanat , M. K. Smith , K. A. Mirica , Sensors 2017, 17, 2192. 10.3390/s17102192 PMC5677178 28946624

[25] M. K. Smith , K. E. Jensen , P. A. Pivak , K. A. Mirica , Chem. Mater. 2016, 28, 5264.

[26] M. S. Yao , X. J. Lv , Z. H. Fu , W. H. Li , W. H. Deng , G. D. Wu , G. Xu , Angew. Chem., Int. Ed. 2017, 56, 16510. 10.1002/anie.201709558 29071780

[27] Q. Yang , Q. Xu , H.‐L. Jiang , Chem. Soc. Rev. 2017, 46, 4774. 28621344 10.1039/c6cs00724d

[28] D. Astruc , F. Lu , J. R. Aranzaes , Angew. Chem., Int. Ed. 2005, 44, 7852. 10.1002/anie.200500766 16304662

[29] G. Lu , S. Li , Z. Guo , O. K. Farha , B. G. Hauser , X. Qi , Y. Wang , X. Wang , S. Han , X. Liu , J. S. DuChene , H. Zhang , Q. Zhang , X. Chen , J. Ma , S. C. J. Loo , W. D. Wei , Y. Yang , J. T. Hupp , F. Huo , Nat. Chem. 2012, 4, 310. 22437717 10.1038/nchem.1272

[30] W. Zhang , Y. Liu , G. Lu , Y. Wang , S. Li , C. Cui , J. Wu , Z. Xu , D. Tian , W. Huang , J. S. DuChene , W. D. Wei , H. Chen , Y. Yang , F. Huo , Adv. Mater. 2015, 27, 2923. 25828431 10.1002/adma.201405752

[31] K. M. Choi , K. Na , G. A. Somorjai , O. M. Yaghi , J. Am. Chem. Soc. 2015, 137, 7810. 26023888 10.1021/jacs.5b03540

[32] X. Fang , Q. Shang , Y. Wang , L. Jiao , T. Yao , Y. Li , Q. Zhang , Y. Luo , H. L. Jiang , Adv. Mater. 2018, 30, 1705112. 10.1002/adma.201705112 29315871

[33] H. C. Li , Y. J. Zhang , X. Hu , W. J. Liu , J. J. Chen , H. Q. Yu , Adv. Energy Mater. 2018, 8, 1702734.

[34] R.‐W. Huang , Y.‐S. Wei , X.‐Y. Dong , X.‐H. Wu , C.‐X. Du , S.‐Q. Zang , T. C. Mak , Nat. Chem. 2017, 9, 689. 28644463 10.1038/nchem.2718

[35] S.‐Y. Ding , J. Gao , Q. Wang , Y. Zhang , W.‐G. Song , C.‐Y. Su , W. Wang , J. Am. Chem. Soc. 2011, 133, 19816. 22026454 10.1021/ja206846p

[36] M. Hmadeh , Z. Lu , Z. Liu , F. Gándara , H. Furukawa , S. Wan , V. Augustyn , R. Chang , L. Liao , F. Zhou , E. Perre , V. Ozolins , K. Suenaga , X. Duan , B. Dunn , Y. Yamamto , O. Terasaki , O. M. Yaghi , Chem. Mater. 2012, 24, 3511.

[37] W.‐T. Koo , J.‐S. Jang , S.‐J. Choi , H.‐J. Cho , I.‐D. Kim , ACS Appl. Mater. Interfaces 2017, 9, 18069. 28492302 10.1021/acsami.7b04657

[38] D.‐H. Kim , J.‐S. Jang , W.‐T. Koo , S.‐J. Choi , H.‐J. Cho , M.‐H. Kim , S.‐J. Kim , I.‐D. Kim , ACS Sens. 2018, 3, 1164. 29762012 10.1021/acssensors.8b00210

[39] Y. J. Jeong , W.‐T. Koo , J.‐S. Jang , D.‐H. Kim , M.‐H. Kim , I.‐D. Kim , ACS Appl. Mater. Interfaces 2018, 10, 2016. 29260542 10.1021/acsami.7b16258

[40] K. Mishra , N. Basavegowda , Y. R. Lee , Catal. Sci. Technol. 2015, 5, 2612.

[41] T. L. Tso , E. K. Lee , J. Phys. Chem. 1985, 89, 1612.

[42] H. Liu , M. Li , O. Voznyy , L. Hu , Q. Fu , D. Zhou , Z. Xia , E. H. Sargent , J. Tang , Adv. Mater. 2014, 26, 2718. 24452852 10.1002/adma.201304366

[43] T. W. Hesterberg , W. B. Bunn , R. O. McClellan , A. K. Hamade , C. M. Long , P. A. Valberg , Crit. Rev. Toxicol. 2009, 39, 743. 19852560 10.3109/10408440903294945

[44] V. Rubio‐Gimenez , N. Almora‐Barrios , G. Escorcia‐Ariza , M. Galbiati , M. Sessolo , S. Tatay , C. Marti‐Gastaldo , Angew. Chem., Int. Ed. 2018, 57, 15086. 10.1002/anie.201808242 30238608

[45] H. B. Michaelson , J. Appl. Phys. 1977, 48, 4729.

[46] H. Zhang , S. Pokhrel , Z. Ji , H. Meng , X. Wang , S. Lin , C. H. Chang , L. Li , R. Li , B. Sun , M. Wang , Y.‐P. Liao , R. Liu , T. Xia , L. Madler , A. E. Nel , J. Am. Chem. Soc. 2014, 136, 6406. 24673286 10.1021/ja501699e PMC4410908

[47] W. Liu , L. Xu , K. Sheng , X. Zhou , B. Dong , G. Lu , H. Song , NPG Asia Mater. 2018, 10, 293.

[48] OSHA , Occupational Safety and Health Standards: Permissible Exposure Limits (PELs), Occupational Safety and Health Administration (OSHA), Washington, DC 2014.

[49] K. Rui , X. Wang , M. Du , Y. Zhang , Q. Wang , Z. Ma , Q. Zhang , D. Li , X. Huang , G. Sun , ACS Appl. Mater. Interfaces 2018, 10, 2837. 29286235 10.1021/acsami.7b16761

[50] S.‐J. Choi , H.‐J. Choi , W.‐T. Koo , D. Huh , H. Lee , I.‐D. Kim , ACS Appl. Mater. Interfaces 2017, 9, 40593. 29083142 10.1021/acsami.7b11317

[51] O. Yassine , O. Shekhah , A. H. Assen , Y. Belmabkhout , K. N. Salama , M. Eddaoudi , Angew. Chem., Int. Ed. 2016, 55, 15879. 10.1002/anie.201608780 27797152

[52] Z. Chen , J. Wang , D. Pan , Y. Wang , R. Noetzel , H. Li , P. Xie , W. Pei , A. Umar , L. Jiang , N. Li , N. F. Rooij , G. Zhou , ACS Nano 2018, 12, 2521. 29512386 10.1021/acsnano.7b08294

[53] W. Yang , P. Wan , X. Zhou , J. Hu , Y. Guan , L. Feng , ACS Appl. Mater. Interfaces 2014, 6, 21093. 25399743 10.1021/am505949a

[54] B. Cho , M. G. Hahm , M. Choi , J. Yoon , A. R. Kim , Y.‐J. Lee , S.‐G. Park , J.‐D. Kwon , C. S. Kim , M. Song , Y. Jeong , K.‐S. Nam , S. Lee , T. J. Youu , C. G. Kang , B. H. Lee , H. C. Ko , P. M. Ajayan , D.‐H. Kim , Sci. Rep. 2015, 5, 8052. 25623472 10.1038/srep08052 PMC4307013

[55] K. Y. Ko , J.‐G. Song , Y. Kim , T. Choi , S. Shin , C. W. Lee , K. Lee , J. Koo , H. Lee , J. Kim , T. Lee , J. Park , H. Kim , ACS Nano 2016, 10, 9287. 27666720 10.1021/acsnano.6b03631

[56] T. Xu , Y. Liu , Y. Pei , Y. Chen , Z. Jiang , Z. Shi , J. Xu , D. Wu , Y. Tian , X. Li , Sens. Actuators, B 2018, 259, 789.

[57] J.‐W. Yoon , J.‐S. Kim , T.‐H. Kim , Y. J. Hong , Y. C. Kang , J.‐H. Lee , Small 2016, 12, 4229. 27357165 10.1002/smll.201601507

[58] W. Liu , K. Sheng , X. Zhou , B. Dong , G. Lu , H. Song , NPG Asia Mater. 2018, 10, 293.

[59] J. Z. Ou , W. Ge , B. Carey , T. Daeneke , A. Rotbart , W. Shan , Y. Wang , Z. Fu , A. F. Chrimes , W. Wlodarski , S. P. Russo , Y. X. Li , K. Kalantar‐Zadeh , ACS Nano 2015, 9, 10313. 26447741 10.1021/acsnano.5b04343

[60] C. Y. Lee , M. S. Strano , Langmuir 2005, 21, 5192. 15896070 10.1021/la046867i

[61] S. Roy , A. Baiker , Chem. Rev. 2009, 109, 4054. 19456148 10.1021/cr800496f

[62] D. T. Wickham , B. A. Banse , B. E. Koel , Surf. Sci. 1991, 243, 83.

