# Supplementary material for: Catalytic Metal Nanoparticles Embedded in Conductive Metal–Organic Frameworks for Chemiresistors: Highly Active and Conductive Porous Materials
Source: Adv Sci (Weinh). 2019 Sep 12;6(21):1900250. doi: 10.1002/advs.201900250 (PMC6839632; doi:10.1002/advs.201900250)
Supplement: Supplementary file 1 — Supplementary [file ADVS-6-1900250-s001.pdf]

Copyright WILEY-VCH Verlag GmbH & Co. KGaA, 69469 Weinheim, Germany, 2019.

ADVANCED SCIENCE
Open Access

Supporting Information

for *Adv. Sci.,* DOI: 10.1002/advs.201900250

Catalytic Metal Nanoparticles Embedded in Conductive Metal–Organic Frameworks for Chemiresistors: Highly Active and Conductive Porous Materials

*Won-Tae Koo, Sang-Joon Kim, Ji-Soo Jang, Dong-Ha Kim, and Il-Doo Kim**

Copyright WILEY-VCH Verlag GmbH & Co. KGaA, 69469 Weinheim, Germany, 2019.

Supporting Information

Catalytic Metal Nanoparticles Embedded in Conductive Metal–Organic Frameworks for Chemiresistors: Highly Active and Conductive Porous Materials

*Won-Tae Koo, Sang-Joon Kim, Ji-Soo Jang, Dong-Ha Kim, and Il-Doo Kim**

Table of Contents

- **Figure S1.** EDS elemental mapping images of Pd@Cu_3(HHTP)$_2$ and Pt@Cu_3(HHTP)$_2$.

- **Figure S2.** XPS analysis of Pd@Cu_3(HHTP)$_2$.

- **Figure S3.** XPS analysis of Pt@Cu_3(HHTP)$_2$.

- **Figure S4.** NO sensing properties of the sensors.

- **Figure S5.** Schematic illustration of the fermi levels of the samples.

- **Figure S6.** Dynamic response transitions of the sensors.

- **Figure S7.** Calculated response times of the sensors.

- **Figure S8.** Exponential fitting curves of the sensors operated at room temperature (25 ºC).

- **Figure S9.** Exponential fitting curves of the sensors operated at 50 ºC.

- **Figure S10.** Exponential fitting curves of the sensors operated at 75 ºC.

- **Table S1.** The lattice spacing of the samples calculated from the refinement of XRD data.

- **Table S2.** The summary of recent studies on NO_2 sensors operated at room temperature in air.

- **Table S3.** The summary of the reaction rate constants of the sensors.

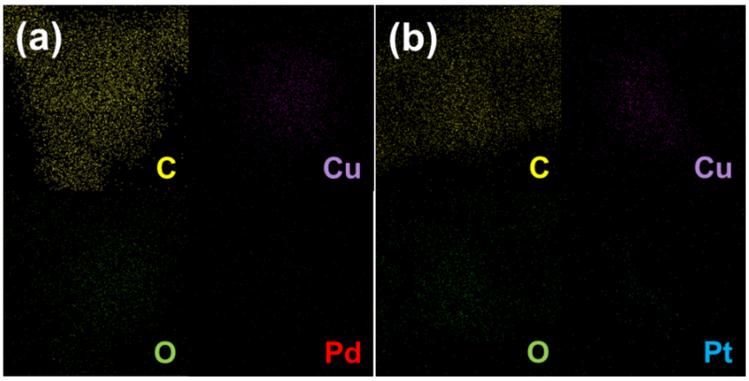

Figure S1. EDS elemental mapping images of (a) Pd@Cu_3(HHTP)$_2$ and Pt@Cu_3(HHTP)$_2$.

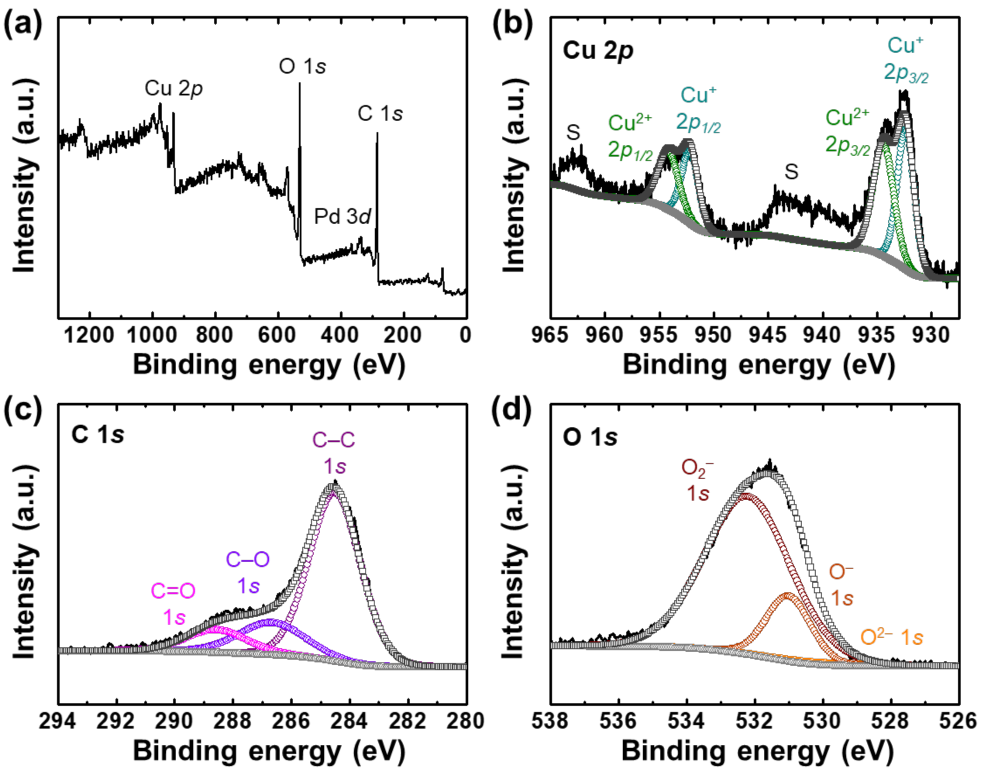

Figure S2. XPS analysis of Pd@$Cu_3(HHTP)_2$: (a) The broad spectra, and the high resolution spectra of (b) Cu 2*p*, (c) C 1*s*, and (d) O 1*s*.

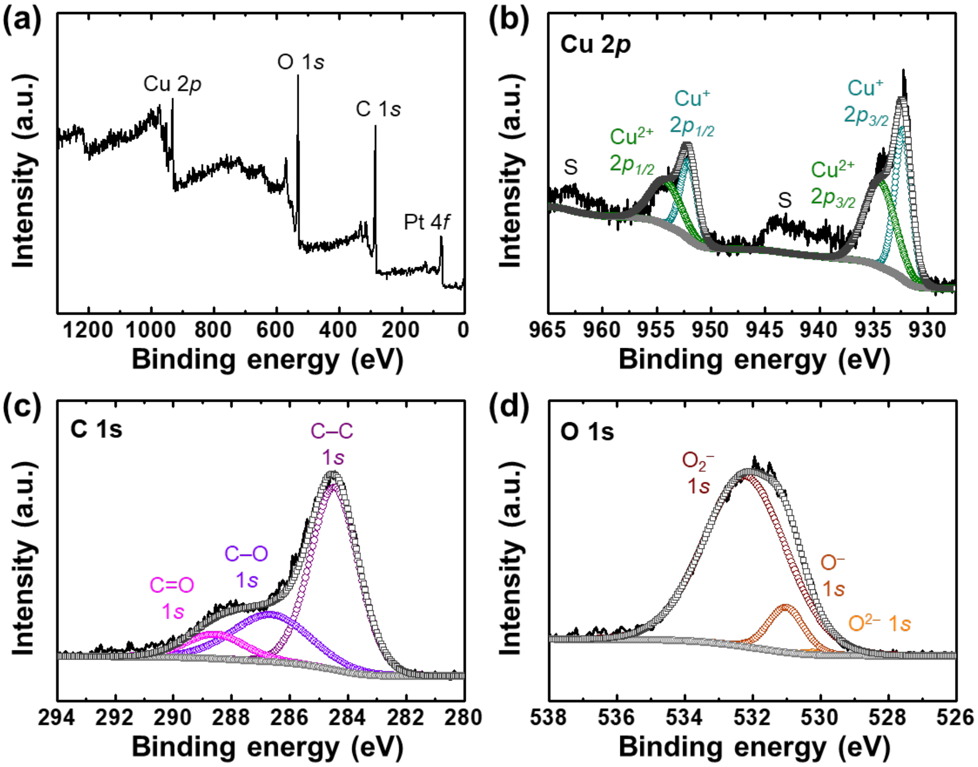

Figure S3. XPS analysis of Pt@$Cu_3(HHTP)_2$: (a) The broad spectra, and the high resolution spectra of (b) Cu 2*p*, (c) C 1*s*, and (d) O 1*s*.

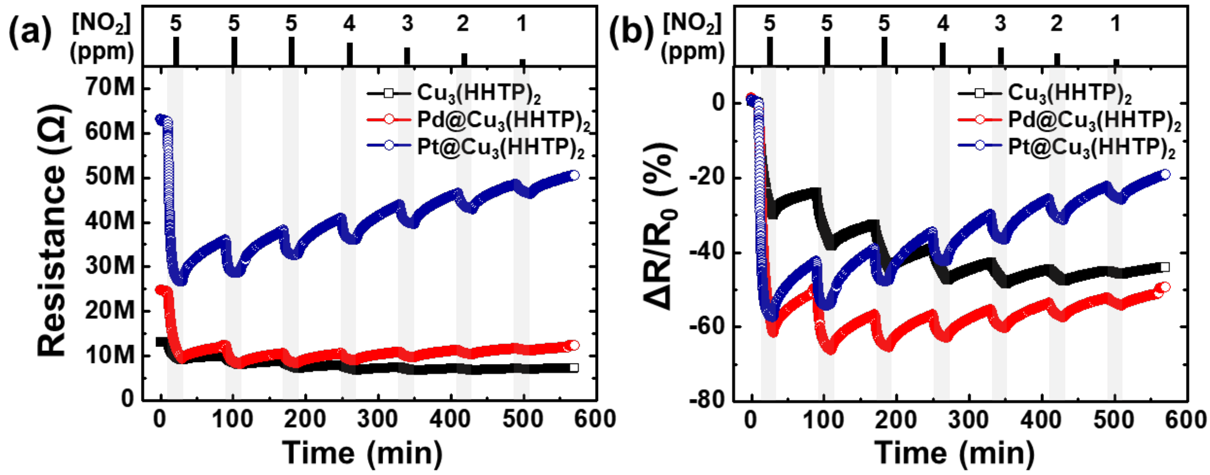

Figure S4. (a) Dynamic resistance transitions of $Cu_3(HHTP)_2$, $Pd@Cu_3(HHTP)_2$, and $Pt@Cu_3(HHTP)_2$ to 1–5 ppm of NO_2 at room temperature in air, and (b) corresponding response variations of the sensors.

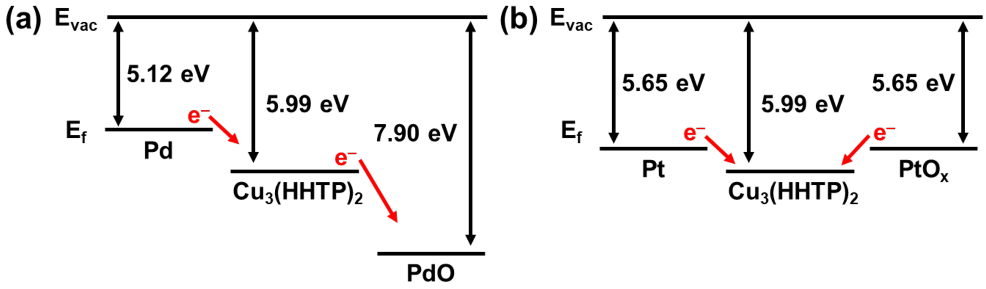

Figure S5. Schematic illustration of the fermi levels of (a) Pd@$Cu_3(HHTP)_2$ and (b) Pt@$Cu_3(HHTP)_2$.

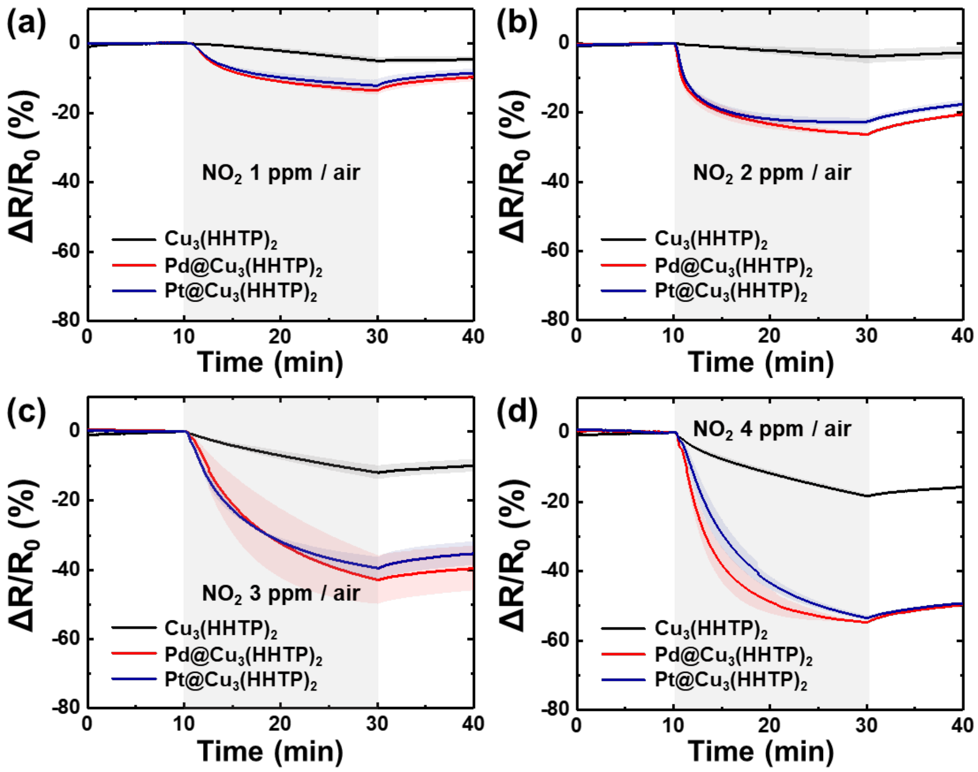

Figure S6. Dynamic response transitions of $Cu_3(HHTP)_2$, Pd@$Cu_3(HHTP)_2$, and Pt@$Cu_3(HHTP)_2$ toward 1–4 ppm of NO_2 in air: (a) 1 ppm, (b) 2 ppm, (c) 3 ppm, and (d) 4 ppm.

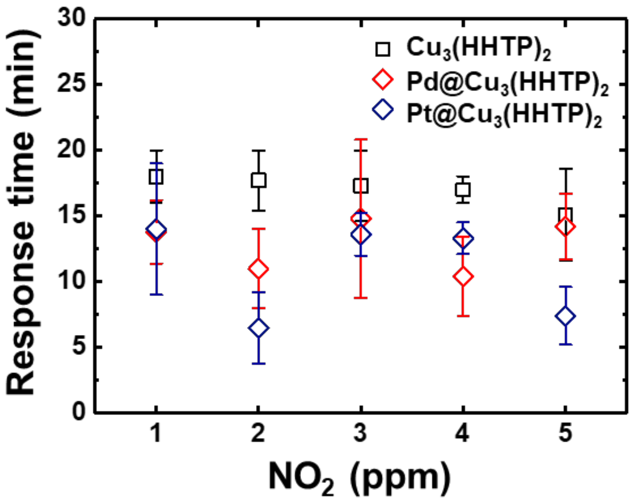

Figure S7. Calculated response times of $Cu_3(HHTP)_2$, $Pd@Cu_3(HHTP)_2$, and $Pt@Cu_3(HHTP)_2$ based sensors to 1–5 ppm of NO_2 at room temperature in air.

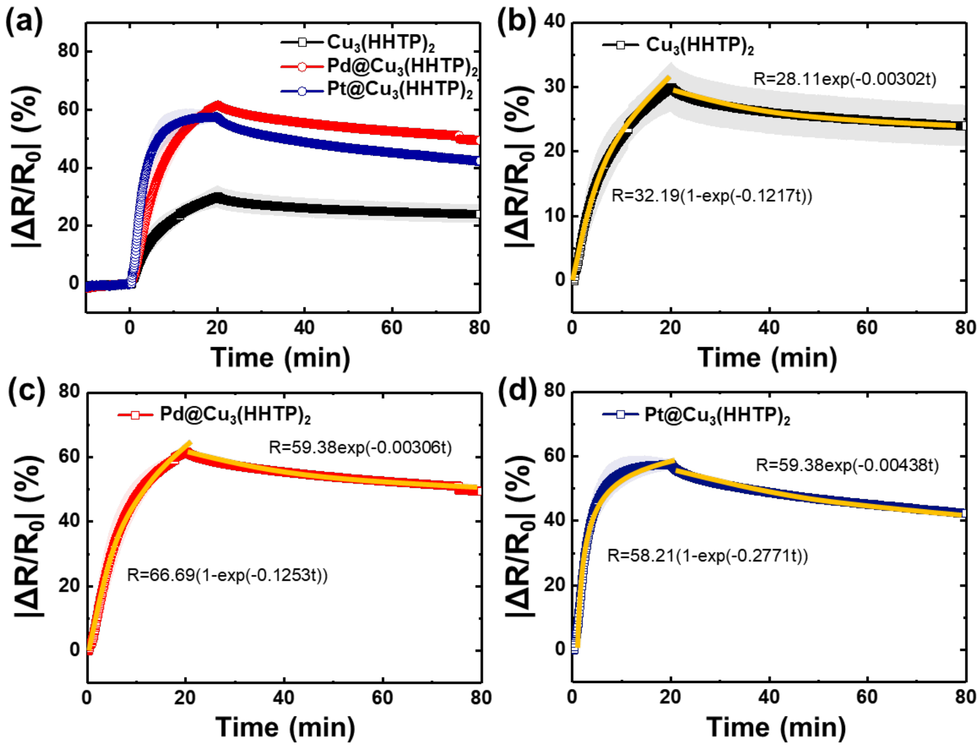

Figure S8. (a) Dynamic response transitions of the sensors to 5 ppm of NO_2 at room temperature (25 ºC). Exponential fitting curves of the sensors by using equation 1 and 2: (b) pristine $Cu_3(HHTP)_2$, (c) $Pd@Cu_3(HHTP)_2$, and (d) $Pt@Cu_3(HHTP)_2$.

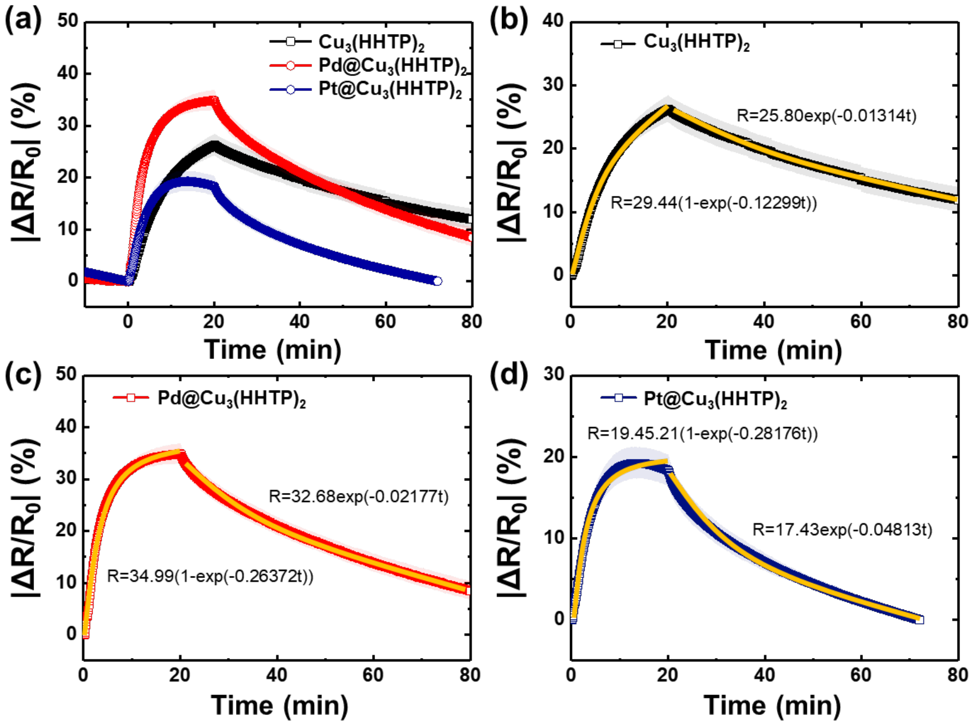

Figure S9. (a) Dynamic response transitions of the sensors to 5 ppm of NO_2 at 50 ºC. Exponential fitting curves of the sensors by using equation 1 and 2: (b) pristine $Cu_3(HHTP)_2$, (c) $Pd@Cu_3(HHTP)_2$, and (d) $Pt@Cu_3(HHTP)_2$.

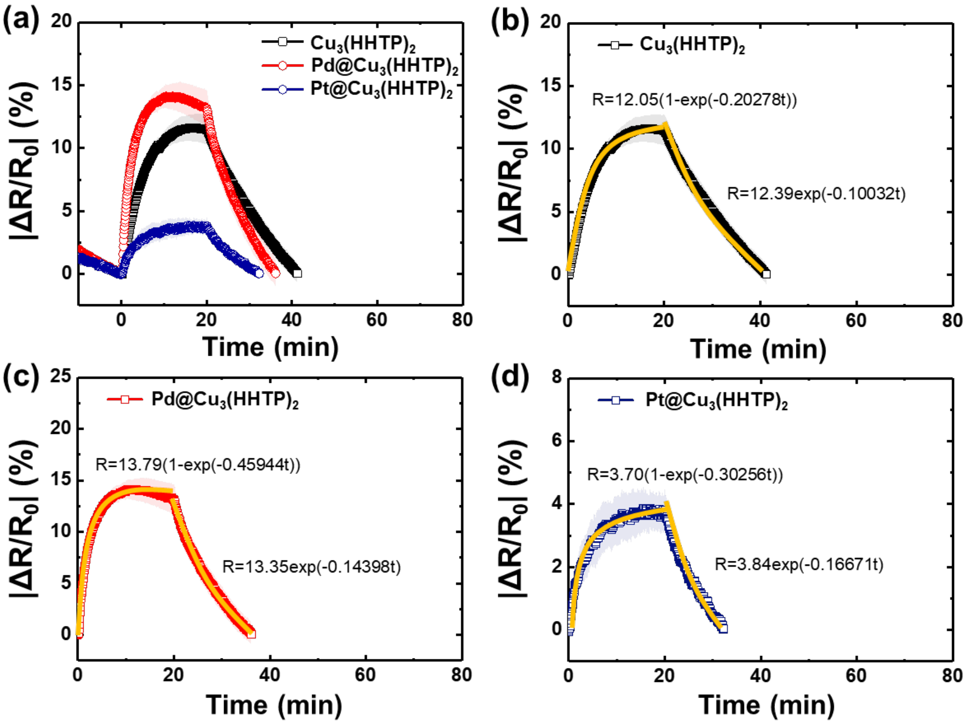

Figure S10. (a) Dynamic response transitions of the sensors to 5 ppm of NO_2 at elevated temperature (75 ºC). Exponential fitting curves of the sensors by using equation 1 and 2: (b) pristine $Cu_3(HHTP)_2$, (c) Pd@$Cu_3(HHTP)_2$, and (d) Pt@$Cu_3(HHTP)_2$.

<Table S1>

Table S1. The lattice spacing of the samples calculated from the refinement of XRD data.

Samples	(004) plane (z-axis)	
	Peak position	Lattice distance
$Cu_3(HHTP)_2$	28.292º	3.1518 ± 0.0092 Å
$Pd@Cu_3(HHTP)_2$	28.280º	3.1531 ± 0.1654Å
$Pt@Cu_3(HHTP)_2$	28.269º	3.1543 ± 0.2775 Å

<Table S2>

Table S2. A summary of recent studies on NO_2 sensors operated at room temperature in air.

Materials	Measurement	Balance gas	Reponse definition	Response	LOD[a] ($[NO_2]$)	Ref.[b]
MOF derived carbon	Resistance	Dry air	R_g/R_a	1% at 5 ppm	100 ppb	48
MOF derived PdO-Co_3O_4	Resistance	Dry air	R_g/R_a	5% at 5 ppm	10 ppm	49
Fumarate-**fcu**-MOF	Capacitance	Dry air	C_g/C_a	0.02% at 10 ppm	100 ppb	50
**Pd@Cu_3(HHTP)$_2$**	Resistance	Dry air	R_g/R_a	62.1% at 5 ppm	1 ppm	**this work**
**Pt@Cu_3(HHTP)$_2$**	Resistance	Dry air	R_g/R_a	57.4% at 5 ppm	1 ppm	**this work**
Scrolled graphene	Resistance	Dry air	R_g/R_a	75% at 5 ppm	1 ppm	51
In_2O_3/reduced graphene oxides	Resistance	Dry air	R_g/R_a	50% at 10 ppm	1 ppm	52
MoS_2 nanosheets	Resistance	Dry air	R_g/R_a	15% at 5 ppm	1.5 ppm	53
WS_2/Ag nanowire	Resistance	Dry air	R_g/R_a	58% at 25 ppm	1 ppm	54
WS_2 nanosheets	Resistance	Dry air	R_g/R_a	68.4% at 5 ppm	100 ppb	55

LOD[a] is the limit of detection of the sensors, and Ref.[b]: the reference number in the manuscript.

<Table S3>

Table S3. The summary of the reaction rate constants of $Cu_3(HHTP)_2$, $Pd@Cu_3(HHTP)_2$, and $Pt@Cu_3(HHTP)_2$. The reaction rate constants of the sensors were calculated from the exponential fitting of response curves upon exposure to 5 ppm of NO_2.

Sample	Operating Temperature	k_{ads} (ppm^{-1} s^{-1})^a	k_{des} (s^{-1})^b	K (k_{ads}/k_{des}, ppm^{-1})^c
$Cu_3(HHTP)_2$	25 °C (298 K)	$2.43 \times 10^{-2} \pm 2.20 \times 10^{-3}$	$5.03 \times 10^{-5} \pm 6.67 \times 10^{-6}$	483.8 ± 19.9
	50 °C (323 K)	$2.46 \times 10^{-2} \pm 1.70 \times 10^{-3}$	$2.69 \times 10^{-4} \pm 2.52 \times 10^{-5}$	91.2 ± 3.8
	75 °C (348 K)	$4.02 \times 10^{-2} \pm 3.50 \times 10^{-3}$	$1.67 \times 10^{-3} \pm 6.33 \times 10^{-5}$	24.1 ± 3.2
$Pd@Cu_3(HHTP)_2$	25 °C (298 K)	$2.51 \times 10^{-2} \pm 7.50 \times 10^{-4}$	$5.10 \times 10^{-5} \pm 1.75 \times 10^{-6}$	491.3 ± 13.4
	50 °C (323 K)	$5.27 \times 10^{-2} \pm 9.50 \times 10^{-4}$	$3.63 \times 10^{-4} \pm 5.00 \times 10^{-6}$	145.1 ± 3.2
	75 °C (348 K)	$9.14 \times 10^{-2} \pm \times 8.35 \times 10^{-3}$	$2.40 \times 10^{-3} \pm 2.08 \times 10^{-5}$	38.1 ± 6.6
$Pt@Cu_3(HHTP)_2$	25 °C (298 K)	$5.54 \times 10^{-2} \pm 2.50 \times 10^{-3}$	$7.30 \times 10^{-5} \pm 5.25 \times 10^{-6}$	758.8 ± 13.7
	50 °C (323 K)	$5.62 \times 10^{-2} \pm 1.90 \times 10^{-3}$	$8.02 \times 10^{-4} \pm 3.62 \times 10^{-5}$	70.1 ± 1.5
	75 °C (348 K)	$6.01 \times 10^{-2} \pm 3.95 \times 10^{-3}$	$2.78 \times 10^{-3} \pm 3.59 \times 10^{-4}$	21.6 ± 2.9

$k_{ads}{}^a$ is a adsorption rate constant, $k_{des}{}^b$ is a desorption rate constant, and K (k_{ads}/k_{des}) is an equilibrium constant.
